# Supplementary material for: Factors Associated with and Prognosis Impact of Perceived Sleep Quality and Estimated Quantity in Patients Receiving Non-Invasive Ventilation for Acute Respiratory Failure
Source: J Clin Med. 2022 Aug 8;11(15):4620. doi: 10.3390/jcm11154620 (PMC9369912; doi:10.3390/jcm11154620)
Supplement: Supplementary file 1 [file jcm-11-04620-s001.zip › jcm-1843261-supplementary.pdf]

Table S1. **Outcome and post-intensive care unit burden according to the sleep quality and sleep duration**

	All patients (n=389)	Poor sleep quality (n=155)	Acceptable-to-very good sleep quality (n=234)	p-value	Short sleep duration (n=148)	Acceptable sleep duration (n=172)	p-value
**ICU discharge**							
ICU length of stay, *days*	5 (3-9)	6 (3-12)	5 (3-8)	0.174	6 (2-12)	5 (3-8)	0.451
ICU mortality, *n (%)* (n=378)	35 (9)	17 (11)	18 (8)	0.278	18 (13)	13 (8)	0.183
**Hospital mortality**, *n (%)* (n=359)	60 (17)	28 (20)	32(15)	0.246	28 (21)	25 (15)	0.222
**90-day mortality**, *n (%)* (n=208)	72 (35)	30 (34)	42 (35)	0.999	31 (36)	34 (37)	0.999
**90-day assessment in surviving patients**							
HADS anxiety at day-90 (n=69)	4 (2-10)	5 (1-9)	4 (2-11)	0.546	4 (1-11)	4 (2-8)	0.790
HADS depression at day-90 (n=69)	6 (2-9)	5 (2-8)	6 (2-10)	0.483	5 (2-8)	6 (1-10)	0.604
IES at day-90 (n=73)	2 (0-11)	2 (0-9)	3 (0-14)	0.452	2 (0-9)	3 (0-10)	0.525

Data are presented as n (%) or median (interquartile range); Short sleep duration < 4 hours; Acceptable sleep duration ≥ 4 hours; BMI: body mass index; SAPS2: Simplified Acute Physiology Score 2; SOFA: Sequential Organ Failure Assessment score; ARF: acute respiratory failure; ICU: intensive care unit; PaO2: arterial oxygen tension; FiO2: inspiratory oxygen fraction; PaCO2: arterial carbon dioxide tension; NIV: noninvasive ventilation. Row n-values are given for perceived sleep quality only.
